# Supplementary material for: rbFOX1/MBNL1 competition for CCUG RNA repeats binding contributes to myotonic dystrophy type 1/type 2 differences
Source: Nat Commun. 2018 May 22;9:2009. doi: 10.1038/s41467-018-04370-x (PMC5964235; doi:10.1038/s41467-018-04370-x)
Supplement: Supplementary file 1 — Supplementary Information [file 41467_2018_4370_MOESM1_ESM.pdf]

Nature Communications

Supplementary Information

rbFOX1/ MBNL1 competition for binding to CCUG RNA repeats may contribute to myotonic dystrophy type 1/ type 2 differences

Chantal Sellier, Estefanía Cerro-Herreros, Markus Blatter, Fernande Freyermuth, Angeline Gaucherot, Frank Ruffenach, Partha Sarkar, Jack Puymirat, Bjarne Udd, John W. Day, Giovanni Meola, Guillaume Bassez, Harutoshi Fujimura, Masanori P. Takahashi, Benedikt Schoser, Denis Furling, Ruben Artero, Frédéric H. T. Allain, Beatriz Llamusi, Nicolas Charlet-Berguerand

Gene name	CUG				CCUG			
	Score	Coverage	Peptides	PSM	Score	Coverage	Peptides	PSM
Terminal uridylyltransferase 4 [TUT4_MOUSE]	67	1	1	21	56	1	1	17
Serine/arginine-rich splicing factor 3 [SRSF3_MOUSE]	23	14	2	8	37	24	3	11
TAR DNA-binding protein 43 [TADBP_MOUSE]	10	9	3	3	37	10	3	9
Heterogeneous nuclear ribonucleoprotein M [HNRPM_MOUSE]	7	3	1	2	20	6	2	6
Protein argonaute-2 OS=Homo sapiens [AGO2_MOUSE]	21	9	5	5	19	8	5	5
Splicing factor 3B subunit 3 [SF3B3_MOUSE]	12	4	3	4	17	6	5	5
**Muscleblind-like protein 1 [MBNL1_MOUSE]**	**30**	**14**	**4**	**10**	**14**	**4**	**2**	**5**
Serine/arginine-rich splicing factor 2 [SRSF2_MOUSE]	8	7	2	2	12	10	2	3
Heterogeneous nuclear ribonucleoprotein U-like 1 [HNRL1_MOUSE]	7	2	1	1	10	4	2	2
U4/U6 small nuclear ribonucleoprotein Prp3 [PRPF3_MOUSE]	3	2	1	1	8	2	1	3
Zinc finger CCCH domain-containing protein 10 [ZC3HA_MOUSE]	6	3	2	2	7	4	1	2
Heterogeneous nuclear ribonucleoprotein D0 [HNRPD_MOUSE]	3	4	1	1	7	7	2	2
snRNA-activating protein complex subunit 1 [SNPC1_MOUSE]	6	5	1	2	7	5	1	2
Heterogeneous nuclear ribonucleoprotein A3 [ROA3_MOUSE]	5	3	2	2	4	5	1	1
**RNA binding protein fox-1 homolog 1 [RFOX1_MOUSE]**					**3**	**4**	**2**	**2**
ATP-dependent RNA helicase DDX25 [DDX25_MOUSE]	3	2	2	1	3	8	1	1
ATP-dependent RNA helicase DDX19A [DD19A_MOUSE]	2	1	1	1	3	4	1	1
Probable ATP-dependent RNA helicase DDX58 [DDX58_MOUSE]	4	3	1	1	3	3	1	1
Poly(rC)-binding protein 2 [PCBP2_MOUSE]	2	1	1	1	3	4	1	1
Nucleolar GTP-binding protein 2 [NOG2_MOUSE]	9	5	2	3	3	3	1	1
Pre-mRNA-splicing factor RBM22 [RBM22_MOUSE]	4	3	1	2	3	4	1	1
ATP-dependent RNA helicase DDX54 [DDX54_MOUSE]	3	2	1	1	3	2	1	1
U3 small nucleolar ribonucleoprotein protein MPP10 [MPP10_MOUSE]	3	2	1	1	3	3	1	1
Double-stranded RNA-specific adenosine deaminase [DSRAD_MOUSE]	3	3	1	1	3	2	1	1
Nuclear RNA export factor 3 [NXF3_MOUSE]	3	3	2	2	3	4	1	1
Exonuclease 3'-5' domain-containing protein 2 [EXD2_MOUSE]	2	1	1	1	3	4	1	1
U2 small nuclear ribonucleoprotein A' [RU2A_MOUSE]	4	2	1	2	3	5	1	1
Pre-rRNA-processing protein TSR2 homolog [TSR2_MOUSE]	2	2	2	1	3	6	1	1
Pre-mRNA-processing factor 40 homolog B [PR40B_MOUSE]	3	1	1	1	2	1	1	1
Nuclear RNA export factor 5 [NXF5_MOUSE]	1	1	1	1	2	2	1	1
Transformer-2 protein homolog alpha [TRA2A_MOUSE]	2	2	1	1	2	4	1	1
RNA-binding protein 44 [RBM44_MOUSE]	2	1	1	1	2	1	1	1
Nucleolysin TIAR [TIAR_MOUSE]	3	3	1	1	2	2	1	1
ATP-dependent RNA helicase DDX39A [DX39A_MOUSE]	2	1	1	1	1	1	1	1

Supplementary Table 1. Identification of C2C12 proteins associated with CCUG repeats.

1 mg of nuclear proteins extracted from 4 days differentiated C2C12 muscle cells was incubated with streptavidin-biotinylated RNA containing either 30 CUG or 30 CCUG repeats, extensively washed, eluted, run on SDS page gel, silver stained, isolated, digested and identified by nano-LC/MS-MS. Contaminants proteins were excluded from the table.

Gene name	CUG Score	CUG Coverage	CUG Peptide	CUG PSM	CCUG Score	CCUG Coverage	CCUG Peptide	CCUG PSM
Terminal uridylyltransferase 4 =Zcchc11 [TUT4_MOUSE]	75	1	1	23	81	1	1	25
Probable ATP-dependent RNA helicase DDX6 OS [DDX6_MOUSE]	84	4	1	17	96	4	1	20
TAR DNA-binding protein 43 =Tardbp [TADBP_MOUSE]	61	23	8	16	76	32	9	18
Poly(rC)-binding protein 2 = Pcbp2 [PCBP2_MOUSE]	44	12	7	11	59	33	7	13
**RNA binding protein fox-1 homolog 2 = Rbfox2 [RFOX2_MOUSE]**					**35**	**14**	**4**	**8**
**RNA binding protein fox-1 homolog 1 = Rbfox1 [RFOX1_MOUSE]**					**33**	**13**	**4**	**8**
**Muscleblind-like protein 1 OS = Mbnl1 [MBNL1_MOUSE]**	**38**	**6**	**3**	**9**	**29**	**5**	**4**	**7**
Protein argonaute-1 = Eif2c1 [AGO1_MOUSE]	21	22	8	6	27	10	6	7
Protein argonaute-2 = Eif2c2 [AGO2_MOUSE]	18	7	5	6	24	9	6	7
Serine/arginine-rich splicing factor 3 = Srsf3 [SRSF3_MOUSE]	11	3	3	4	21	18	3	7
Poly(rC)-binding protein 1 = Pcbp1 [PCBP1_MOUSE]	17	6	2	4	21	13	3	5
Heterogeneous nuclear ribonucleoprotein L = Hnrnpl [HNRPL_MOUSE]	12	5	3	4	18	5	2	5
Heterogeneous nuclear ribonucleoprotein M = Hnrnpm [HNRPM_MOUSE]	19	9	5	4	10	5	3	3
**RNA binding protein fox-1 homolog 3 = Rbfox3 [RFOX3_MOUSE]**					**12**	**10**	**3**	**3**
Nucleolysin TIAR = Tial1 [TIAR_MOUSE]	8	6	2	2	9	8	2	2
Zinc finger CCCH domain-containing protein 10 = Zc3h10 [ZC3HA_MOUSE]	8	6	2	2	9	8	2	2
Far upstream element-binding protein = Khsrp [FUBP2_MOUSE]	3	1	1	1	8	6	2	2
Heterogeneous nuclear ribonucleoprotein U-like 2 = [HNRL2_MOUSE]	3	1	1	1	7	4	2	2
ATP-dependent RNA helicase DDX3Y = Ddx3y [DDX3Y_MOUSE]	3	3	1	1	7	3	2	2
Serine/arginine-rich splicing factor 2 = Srsf2 [SRSF2_MOUSE]	6	9	2	2	6	8	2	2
Heterogeneous nuclear ribonucleoprotein K = Hnrnpk [HNRPK_MOUSE]	3	5	1	1	6	6	2	2
Nucleolin = Ncl [NUCL_MOUSE]	3	4	1	1	6	5	2	2
Serine/arginine-rich splicing factor 5 = Srsf5 [SRSF5_MOUSE]	3	4	1	1	6	3	1	2
Heterogeneous nuclear ribonucleoproteins A2/B1 = [ROA2_MOUSE]	3	9	1	1	6	3	1	2
RNA-binding protein Raly = Raly [RALY_MOUSE]	3	10	1	1	3	12	1	1
Pre-mRNA-splicing factor RBM22 = Rbm22 [RBM22_MOUSE]	3	5	1	1	3	8	1	1
Pre-mRNA-splicing factor 38B = Prpf38b [PR38B_MOUSE]	3	4	1	1	3	6	1	1
U4/U6 small nuclear ribonucleoprotein Prp31 = Prpf31 [PRP31_MOUSE]	3	4	1	1	3	4	1	1
CUGBP Elav-like family member 2 = Celf2 [CELF2_MOUSE]	3	1	1	1	3	2	1	1
Heterogeneous nuclear ribonucleoprotein U-like 1 = [HNRL1_MOUSE]	3	9	1	1	3	2	1	1
Splicing factor, proline- and glutamine-rich = Sfpq [SFPQ_MOUSE]	3	3	1	1	3	2	1	1
Matrin-3 OS = Matr3 [MATR3_MOUSE]	3	1	1	1	3	2	1	1
Fragile X mental retardation protein 1 homolog = Fmr1 [FMR1_MOUSE]	3	3	1	1	2	1	1	1
RNA-binding protein = Rbm10 [RBM10_MOUSE]	3	7	1	1	2	1	1	1
Putative ATP-dependent RNA helicase DHX57 = Dhx57 [DHX57_MOUSE]	2	2	1	1	2	1	1	1

Supplementary Table 2. Identification of brain proteins associated with CCUG repeats.

1 mg of nuclear proteins extracted from adult C57/BL6 wild type mouse brain was incubated with streptavidin-biotinylated RNA containing either 30 CUG or 30 CCUG repeats, extensively washed, eluted, run on SDS page gel, silver stained, isolated, digested and identified by nano-LC/MS-MS.

Contaminants proteins were excluded from the table.

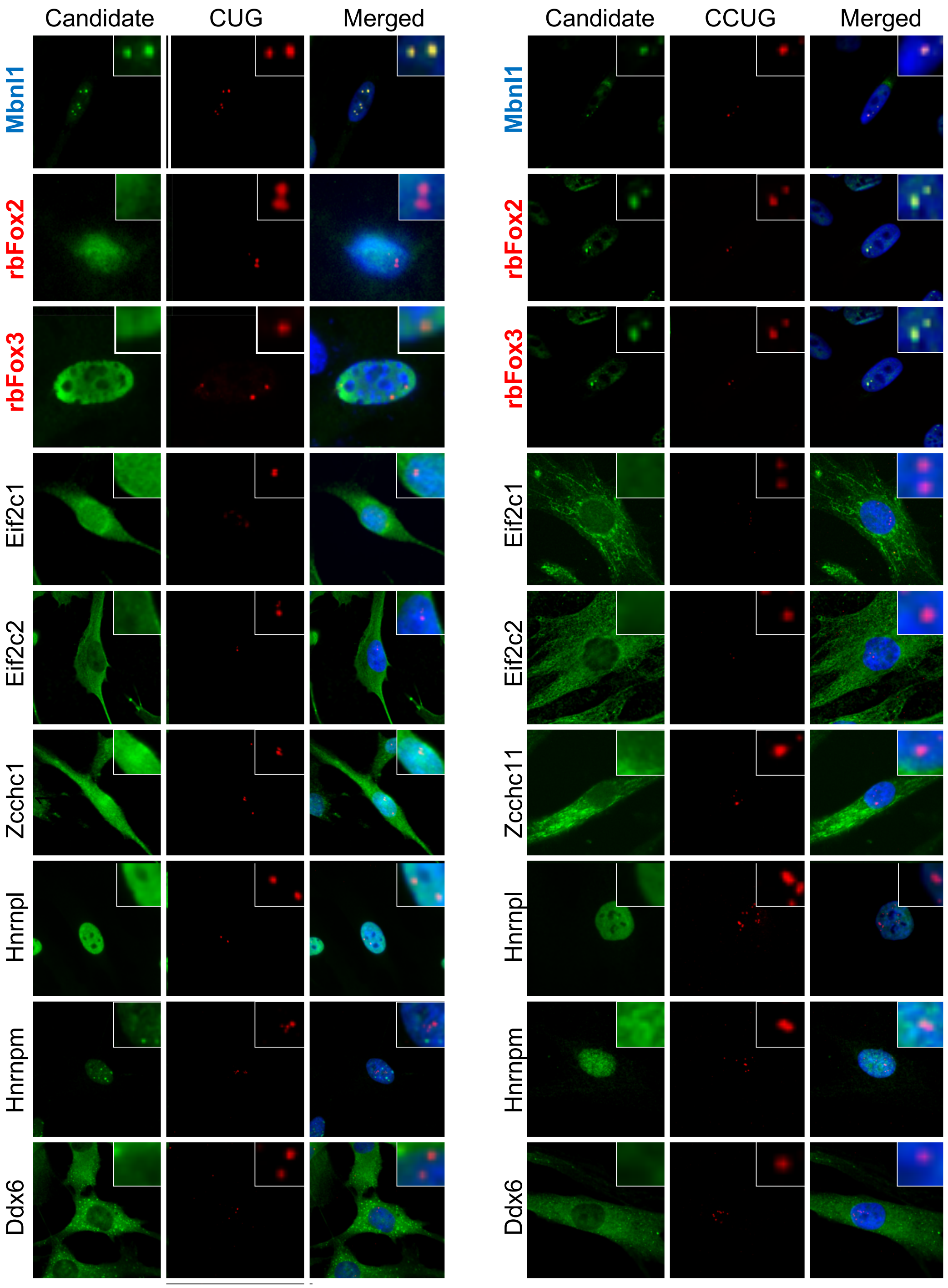
Candidate
CUG
Merged
Candidate
CCUG
Merged
Mbnl1
rbFox2
rbFox3
Eif2c1
Eif2c2
Zcchc1
Hnrnpl
Hnrnpm
Ddx6
Mbnl1
rbFox2
rbFox3
Eif2c1
Eif2c2
Zcchc11
Hnrnpl
Hnrnpm
Ddx6

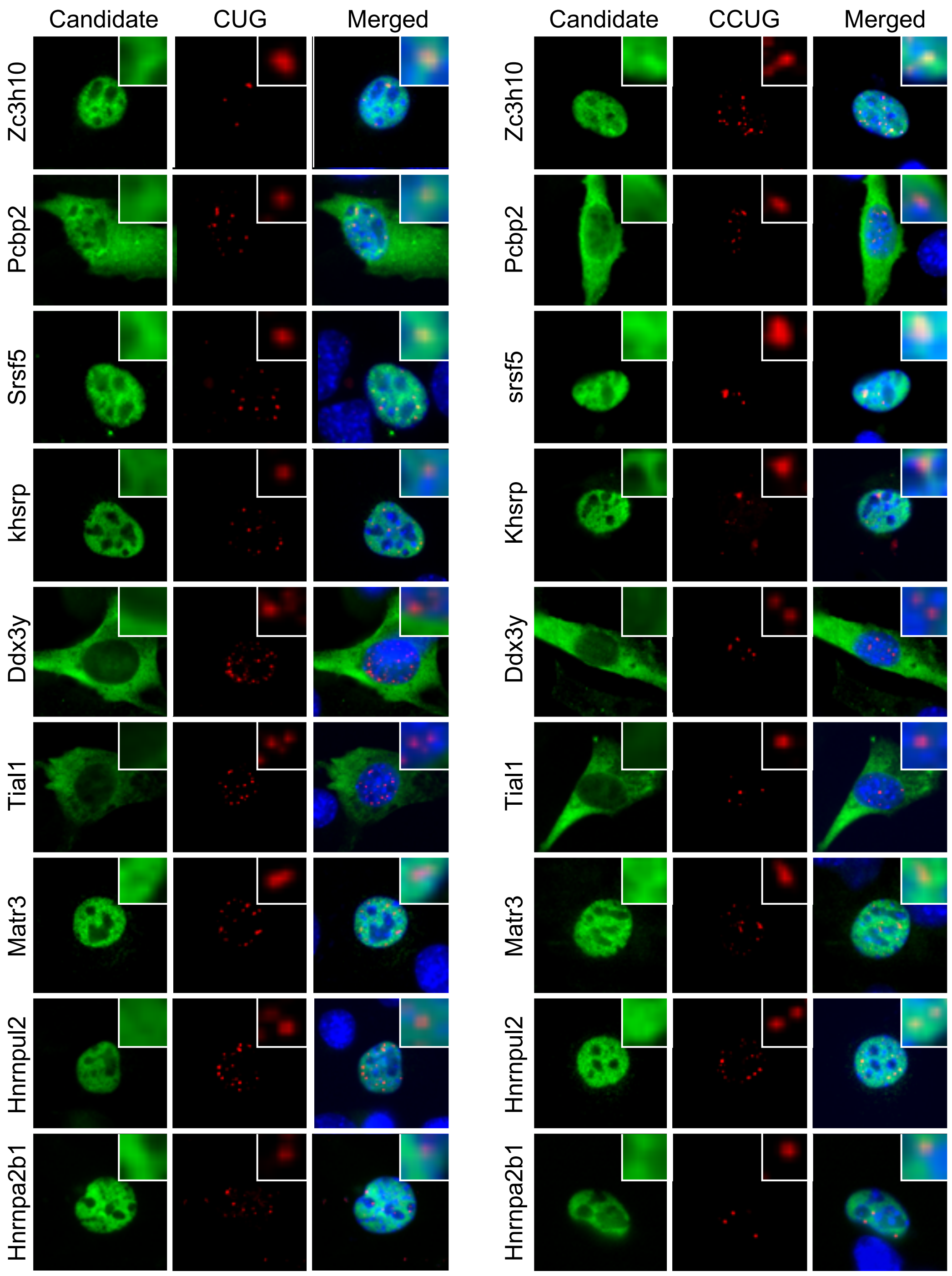

Candidate
CUG
Merged
Candidate
CCUG
Merged
Zc3h10
Pcbp2
Srsf5
khsrp
Ddx3y
Tial1
Matr3
Hnrnpul2
Hnrnpa2b1
Zc3h10
Pcbp2
srsf5
Khsrp
Ddx3y
Tial1
Matr3
Hnrnpul2
Hnrnpa2b1

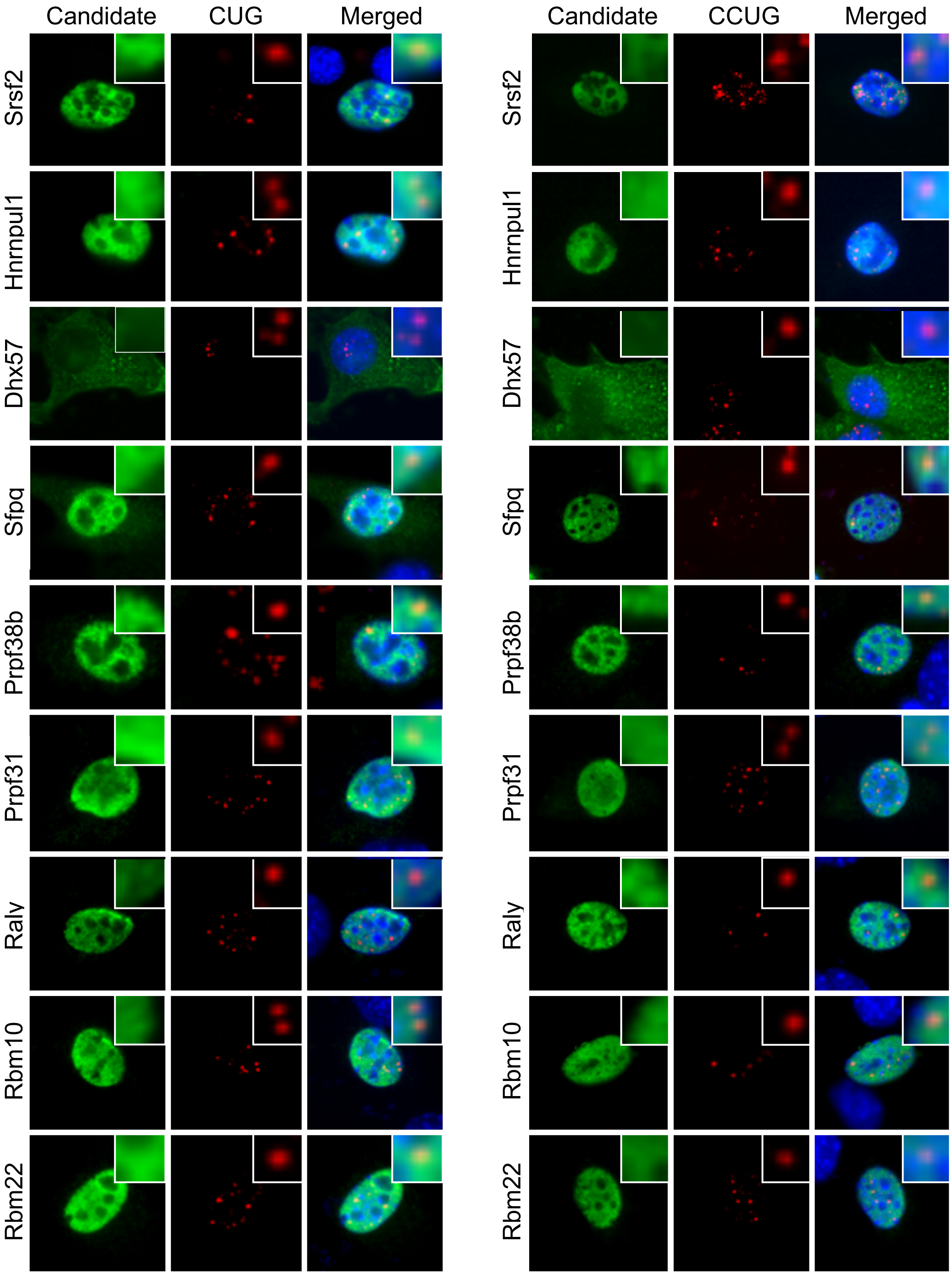

Candidate
CUG
Merged
Candidate
CCUG
Merged
Srsf2
Hnrnpul1
Dhx57
Sfpq
Prpf38b
Prpf31
Raly
Rbm10
Rbm22

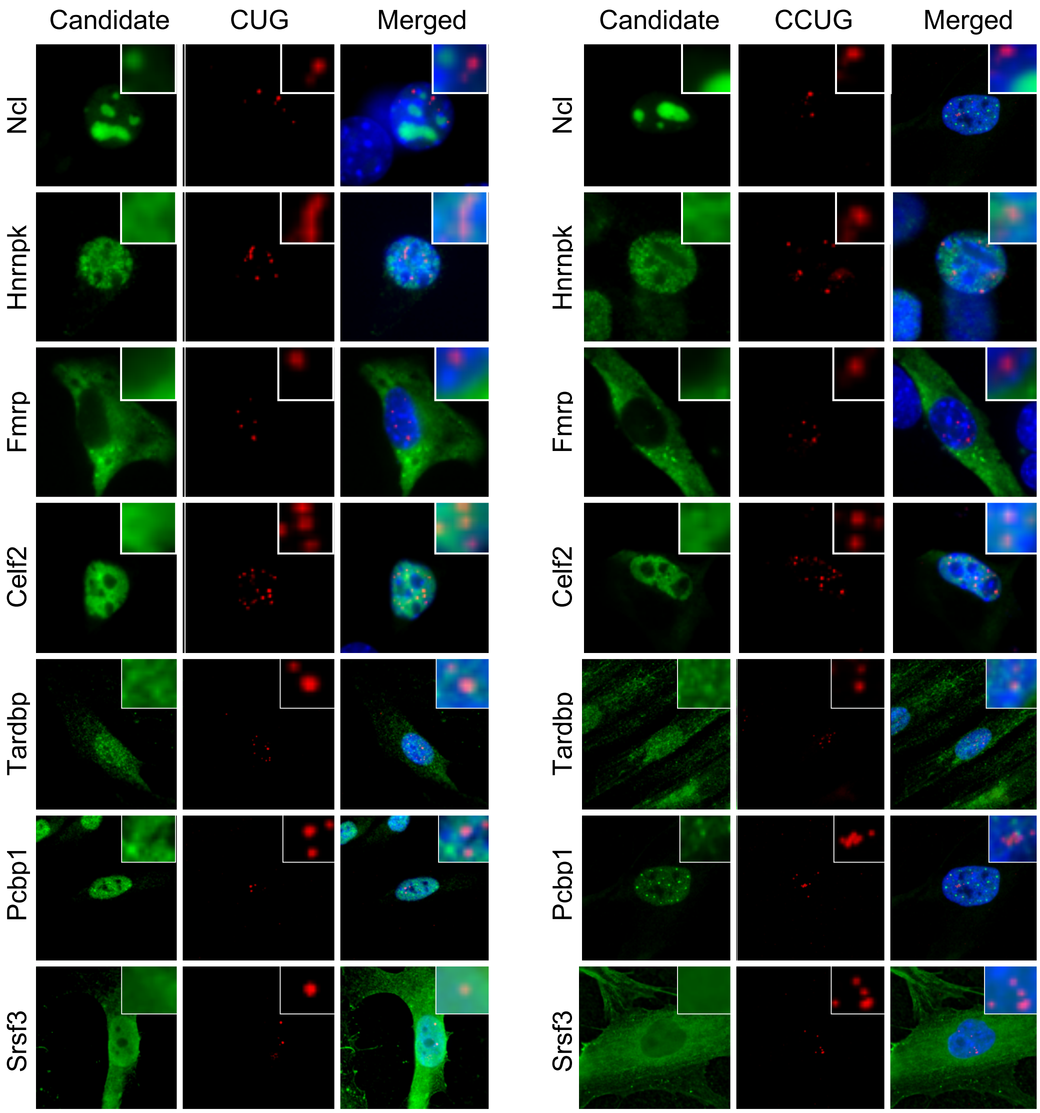

Supplementary Figure 1. Validation of the proteins associated to CCUG repeats.

Candidate proteins found *in vitro* to associate with expanded CUG and/or CCUG repeats were tested for co-localization with RNA foci of expanded CUG or CCUG repeats. C2C12 cells were transfected with a plasmid expressing either 960 CUG repeats or 1,000 CCUG repeats and differentiated during two days and then assayed by RNA FISH using either a CAG8x-Cy3 or a CAGG8x-Cy3 DNA probe followed by immunofluorescence against the protein of interest. Nuclei are labeled by DAPI staining. Magnifications, 630x.

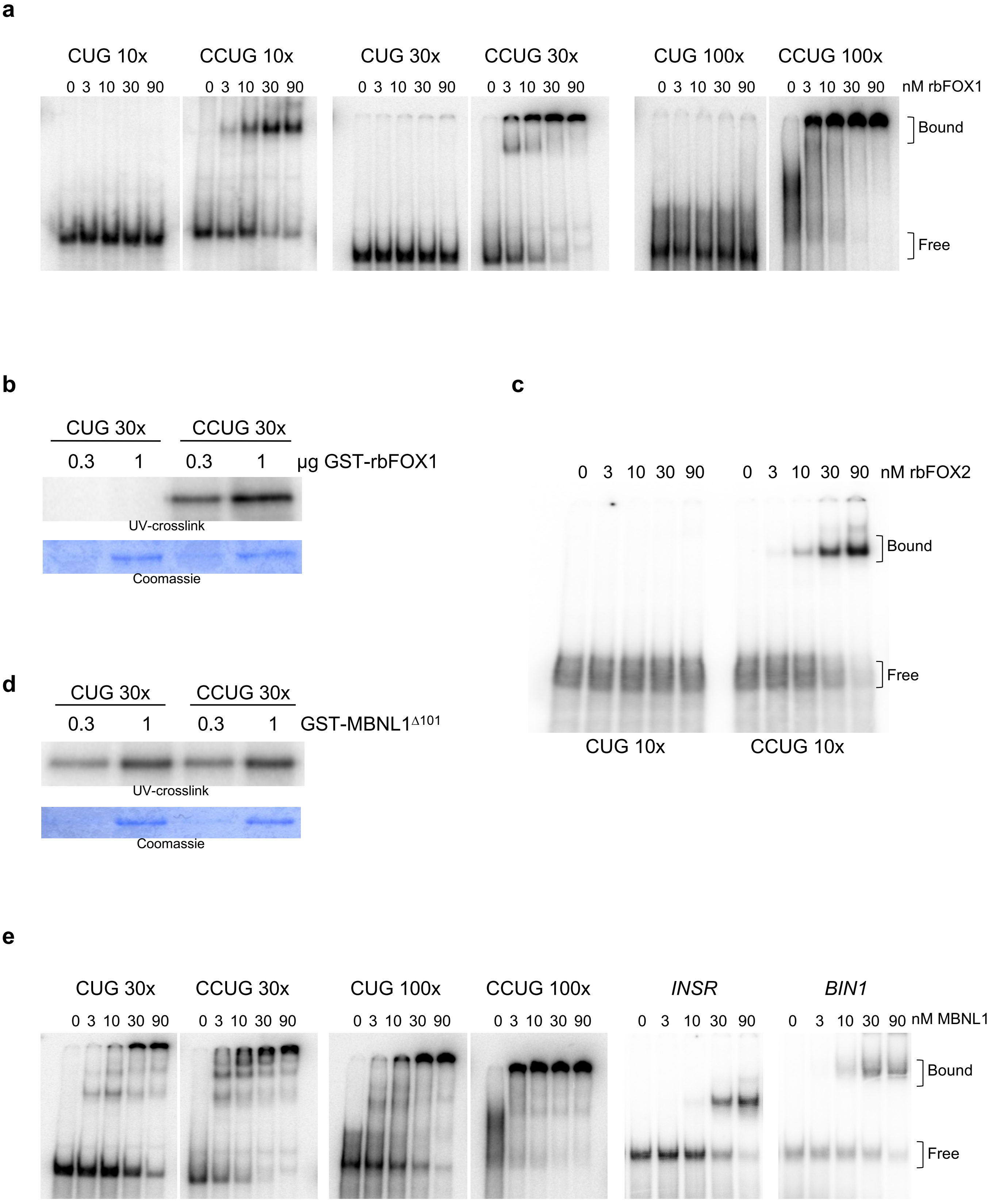
a
CUG 10x
CCUG 10x
CUG 30x
CCUG 30x
CUG 100x
CCUG 100x
0 3 10 30 90
nM rbFOX1
Bound
Free
b
CUG 30x
CCUG 30x
0.3 1 0.3 1
µg GST-rbFOX1
UV-crosslink
Coomassie
c
0 3 10 30 90
0 3 10 30 90 nM rbFOX2
Bound
Free
CUG 10x
CCUG 10x
d
CUG 30x
CCUG 30x
0.3 1 0.3 1
GST-MBNL1Δ101
UV-crosslink
Coomassie
e
CUG 30x
CCUG 30x
CUG 100x
CCUG 100x
INSR
BIN1
0 3 10 30 90
nM MBNL1
Bound
Free

f

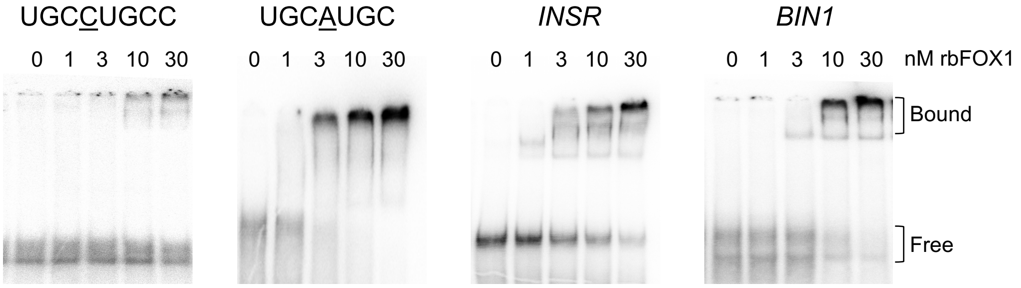

g

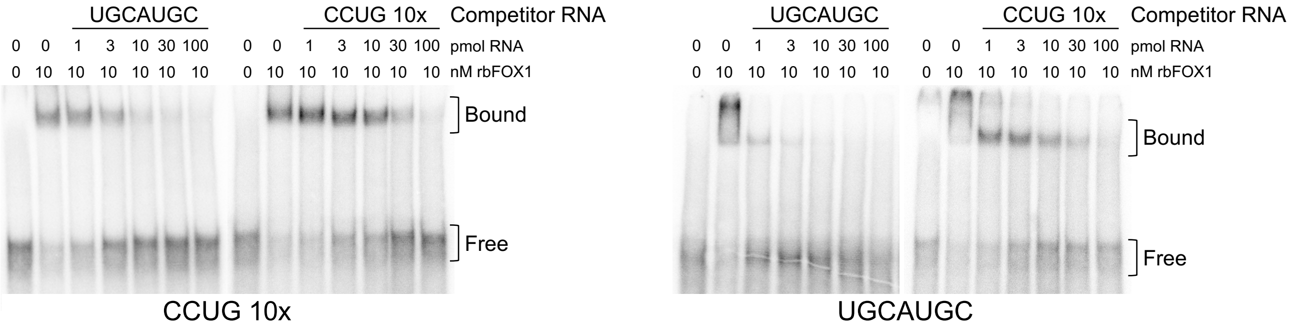

h

	rbFOX1	MBNL1
CUG 10x	No binding	11.7 +- 2.1
CUG 30x	No binding	8.4 +- 1.6
CUG 100x	No binding	4.6 +- 1.2
CCUG 10x	9.7 +- 1.4	9.3 +- 1.7
CCUG 30x	6.3 +- 1.8	6.1 +- 1.3
CCUG 100x	3.2 +- 1.3	2.6 +- 1.0
UGCCUGC	103.7 +- 8.7	37.2 +- 3.7
UGCAUGC	2.3 +- 0.7	43.7 +- 2.4
BIN1	5.8 +- 1.2	17.6 +- 2.1
INSR	4.5 +- 1.5	21.8 +- 3.2
TNNT2	No binding	16.3 +- 2.3
CLCN1	No binding	15.5 +- 2.8

i

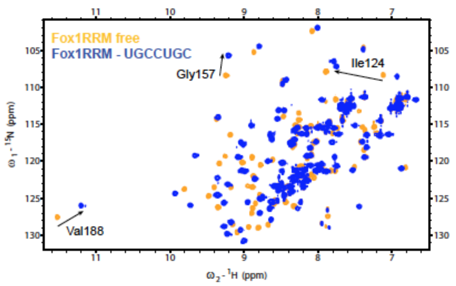

j

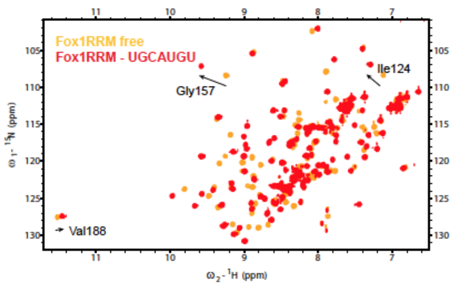

Supplementary Figure 2. rbFOX1 and rbFOX2 bind to expanded CCUG repeats.

a Gel-shift assays of 0, 3, 10, 30 and 90 nM (~2, 6, 20 and 60 ng) of purified recombinant GST-rbFOX1 with 10 pM (3,000 CPM) of uniformly $[\alpha P^{32}]$ internally labeled *in vitro* transcribed RNAs containing either 10, 30 or 100 CUG or CCUG repeats. **b** UV-crosslinking binding assays with 0.5 and 1.5 µM (~0.3 and 1 µg) of purified recombinant GST-tagged rbFOX1 with 100 pM (30,000 CPM) of uniformly $[\alpha P^{32}]$ internally labeled *in vitro* transcribed RNAs containing 30 CUG or CCUG repeats. Upper panel, phosphorImager exposition. Lower panel, control loading of proteins visualized by coomassie staining. **c** Gel-shifts as in **a** but with purified recombinant GST-rbFOX2 binding to 10 CUG or CCUG repeats. **d** UV-crosslinking as in **b** but with 0.3 and 1 µM of purified recombinant GST-tagged MBNL1$^{\Delta 101}$. **e** Gel-shifts as in **a** but with purified recombinant GST-MBNL1$^{\Delta 101}$ binding to 30 or 100 CUG or CCUG repeats or to *INSR* or *BIN1* exon 11 bordering intronic regions. **f** Gel-shifts as in **a** but with GST-rbFOX1 binding to the UGCAUGC or UGCCUGC RNA sequence or to *INSR* or *BIN1* exon 11 bordering intronic regions. **g** Left panel, gel-shift competition of 10 nM of GST-rbFOX1 bound to 30 pmol of $[\alpha P^{32}]$ labeled (CCUG)10x RNA competed with 1, 3, 10, 30 and 100 pmol of unlabeled UGCAUGC or (CCUG)10x RNA. Right panel, gel-shift competition of 10 nM of GST-rbFOX1 bound to 1 pmol of $[\alpha P^{32}]$ labeled UGCAUGC RNA competed with 1, 3, 10, 30 and 100 pmol of unlabeled UGCAUGC or (CCUG)10x RNAs. **h** Apparent KD of the binding of GST-rbFOX1 and GST-MBNL1$^{\Delta 101}$ to the various RNA studied. S.e.m. of five independent experiments. **i-j** Overlay of 15N-1H HSQC spectra of rbFOX1-RRM in free state (yellow) and bound to UGCCUGC (blue) or UGCAUGU (red) RNA. Representative resonances that are perturbed differently comparing these two complexes are labeled and indicated by an arrow and are located around the adenine to cytosine substitution in position 4. These perturbations can be attributed to the different chemical environment caused by the two base types.

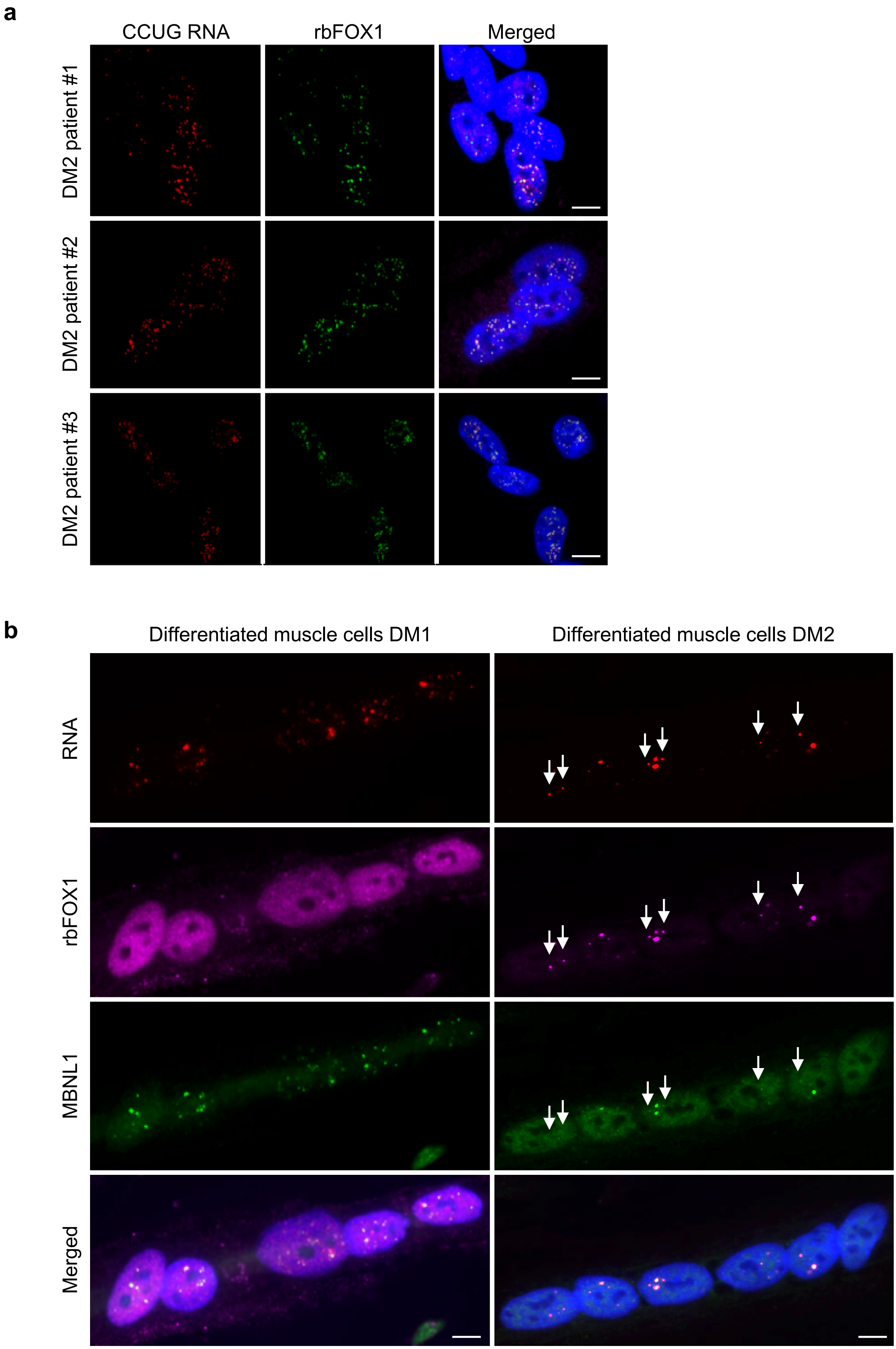
a
CCUG RNA
rbFOX1
Merged
DM2 patient #1
DM2 patient #2
DM2 patient #3
b
Differentiated muscle cells DM1
Differentiated muscle cells DM2
RNA
rbFOX1
MBNL1
Merged

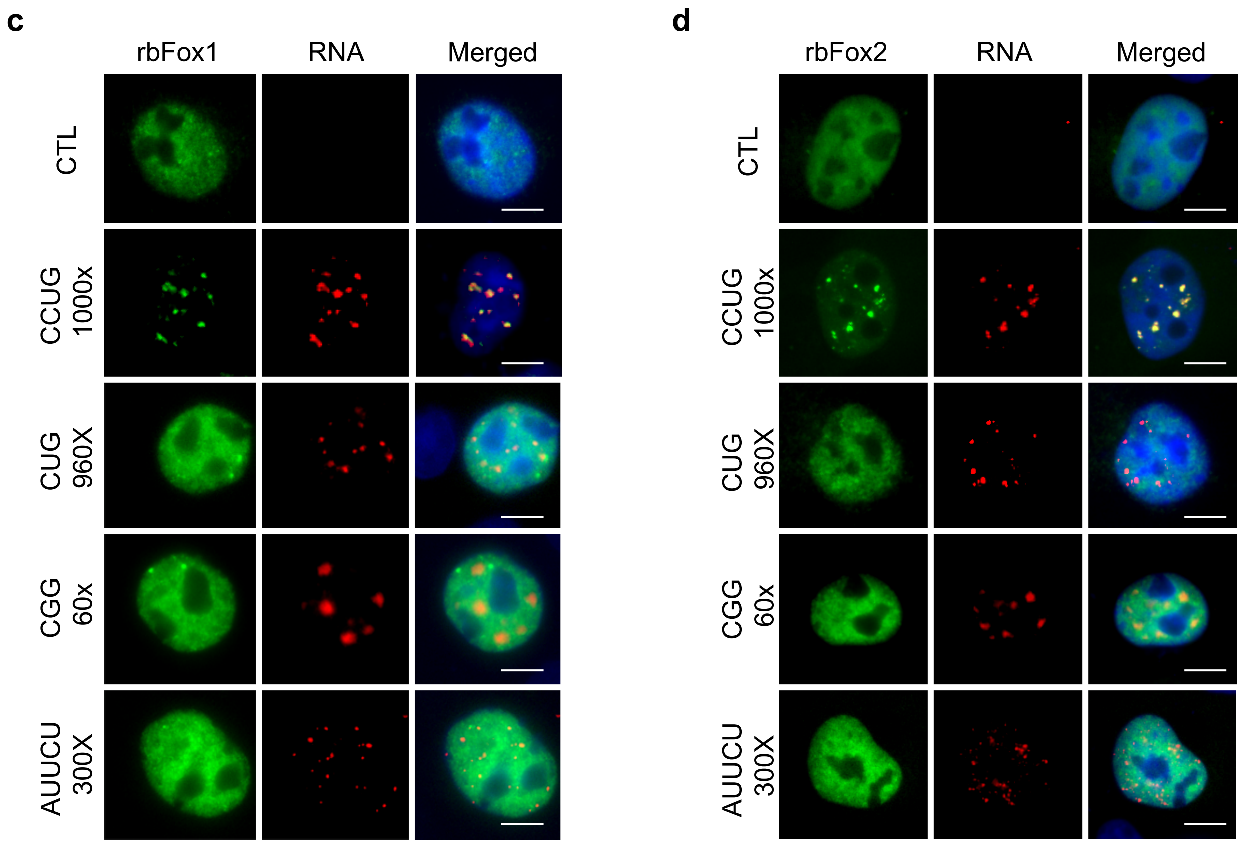

Supplementary Figure 3. rbFOX1 localizes within CCUG foci.

a Representative confocal images of RNA FISH against CCUG repeats coupled to immunofluorescence against rbFOX1 on primary muscle cells differentiated two days and originating from needle muscle biopsies of three different adult individuals with DM2. **b** CUG or CCUG RNA FISH combined to immunofluorescence against MBNL1 and rbFOX1 on six days differentiated muscle cells originating from muscle biopsies of individuals with either DM1 or DM2. Of interest, at two days of differentiation all CCUG RNA foci are positive for MBNL1. In contrast, at six days of differentiation some rare RNA foci of expanded CCUG repeats are negative for MBN1 staining, while positive for rbFOX1 (white arrows). This correlates with the increase expression of rbFOX1 during muscle cell differentiation and may suggest some competition between rbFOX1 and MBNL1 to bind to expanded CCUG RNA repeats. **c** RNA FISH against RNA repeats, coupled to immunofluorescence against endogenous rbFox1 on C2C12 cells transfected with a plasmid expressing either no repeats (CTL), 1,000 CCUG repeats, 960 CUG repeats, 60 CGG repeats or 300 AUUCU repeats, differentiated 48 hours and analyzed by RNA FISH coupled to immunofluorescence using an antibody directed against rbFox1. **d** RNA FISH/ immunofluorescence as in **c** but with an antibody directed against endogenous rbFox2. Magnifications, 630x. Scale bars, 10 μm. Nuclei were counterstained with DAPI.

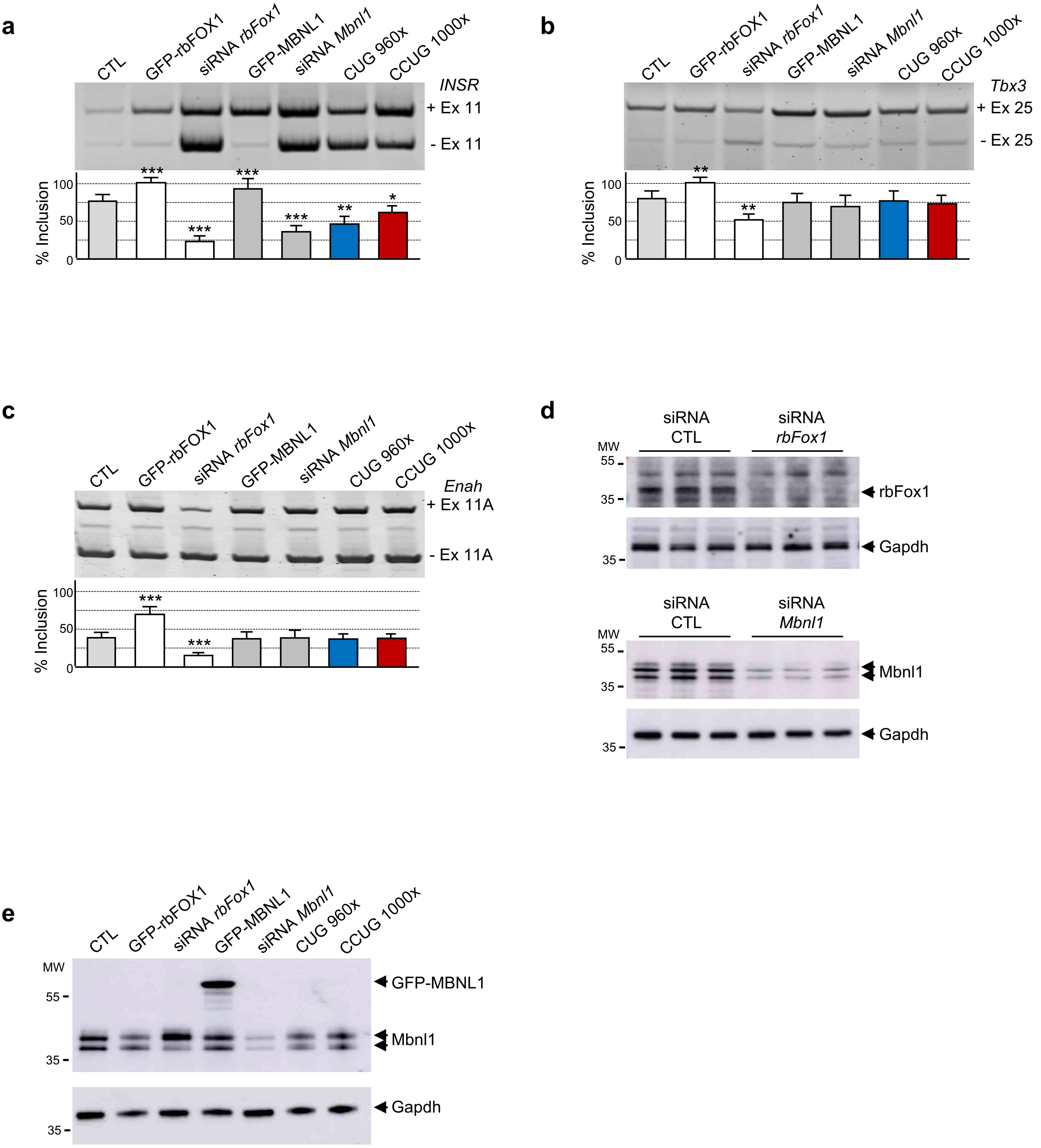
a
CTL
GFP-rbFOX1
siRNA rbFox1
GFP-MBNL1
siRNA Mbnl1
CUG 960x
CCUG 1000x
INSR
+ Ex 11
- Ex 11
% Inclusion
0
50
100
***
***
***
***
**
*
b
CTL
GFP-rbFOX1
siRNA rbFox1
GFP-MBNL1
siRNA Mbnl1
CUG 960x
CCUG 1000x
Tbx3
+ Ex 25
- Ex 25
% Inclusion
0
50
100
**
**
c
CTL
GFP-rbFOX1
siRNA rbFox1
GFP-MBNL1
siRNA Mbnl1
CUG 960x
CCUG 1000x
Enah
+ Ex 11A
- Ex 11A
% Inclusion
0
50
100
***
***
d
siRNA CTL
siRNA rbFox1
MW
55
35
rbFox1
35
Gapdh
siRNA CTL
siRNA Mbnl1
MW
55
35
Mbnl1
35
Gapdh
e
CTL
GFP-rbFOX1
siRNA rbFox1
GFP-MBNL1
siRNA Mbnl1
CUG 960x
CCUG 1000x
MW
55
35
GFP-MBNL1
Mbnl1
35
Gapdh

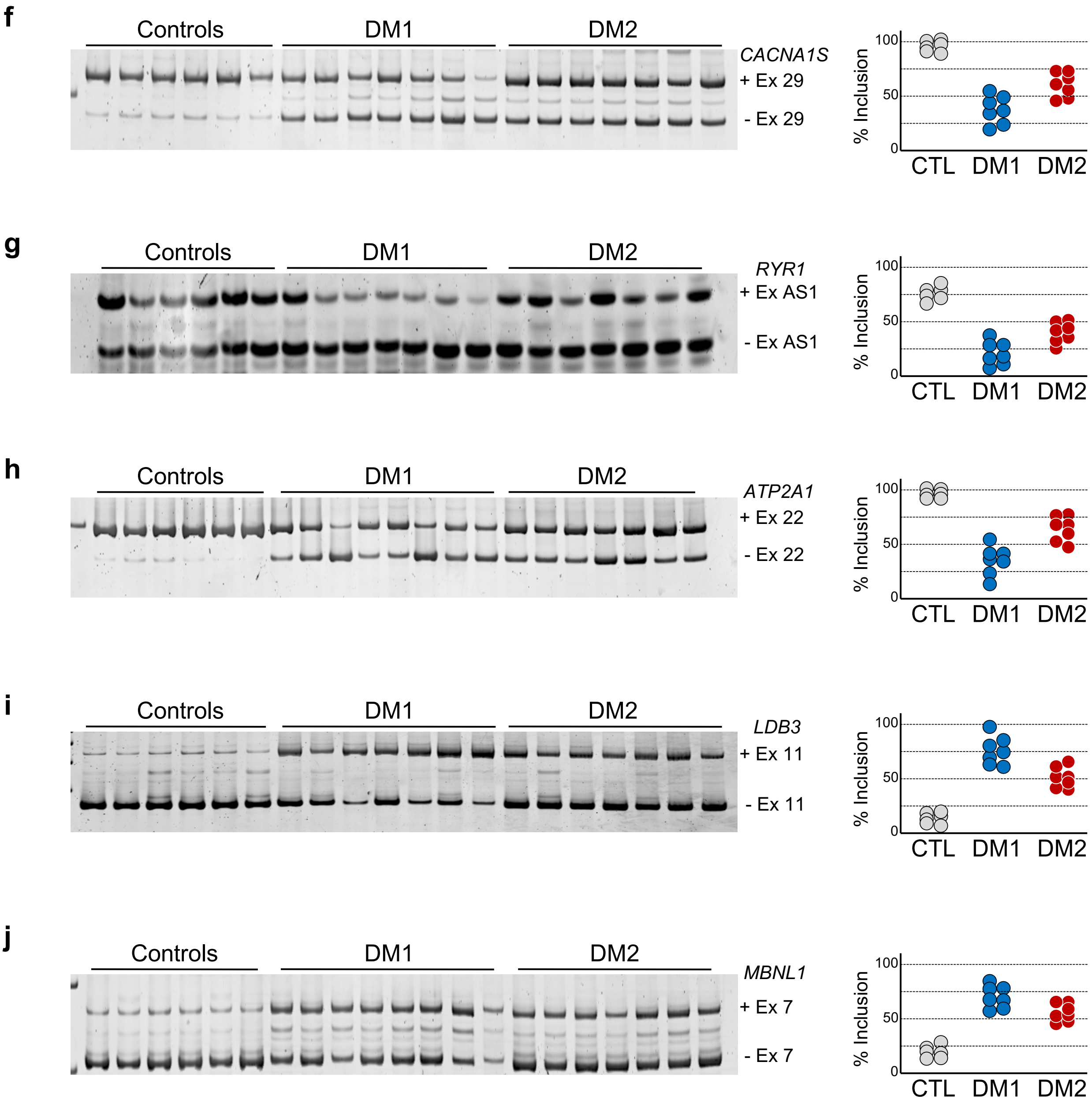
f
Controls
DM1
DM2
CACNA1S
+ Ex 29
- Ex 29
% Inclusion
100
50
0
CTL
DM1
DM2
g
Controls
DM1
DM2
RYR1
+ Ex AS1
- Ex AS1
% Inclusion
100
50
0
CTL
DM1
DM2
h
Controls
DM1
DM2
ATP2A1
+ Ex 22
- Ex 22
% Inclusion
100
50
0
CTL
DM1
DM2
i
Controls
DM1
DM2
LDB3
+ Ex 11
- Ex 11
% Inclusion
100
50
0
CTL
DM1
DM2
j
Controls
DM1
DM2
MBNL1
+ Ex 7
- Ex 7
% Inclusion
100
50
0
CTL
DM1
DM2

Supplementary Figure 4. rbFOX proteins are not sequestered within CCUG RNA foci.

a RT-PCR analysis (upper panel) and quantification (lower panel) of RNA extracted from C2C12 cells co-transfected with a minigene expressing the exon 11 of the insulin receptor *INSR* gene and either with a plasmid expressing rbFOX1, MBNL1, 960 CUG repeats or 1,000 CCUG repeats or with a siRNA directed against *rbFox1* or *Mbnl1*. C2C12 cells were differentiated two days before RNA extraction and RT-PCR analysis of the splicing of the *INSR* minigene. **b-c** As in **a** but without minigene transfection and with RT-PCR analysis of endogenous *Tbx3* and *Enah* pre-mRNAs splicing. **d** Immunoblotting against either rbFox1 or Mbnl1 endogenous expression demonstrates correct siRNA-mediated depletion. **e** Immunoblot against endogenous and exogenous Mbnl1 expression from GFP-FACS sorted C2C12 cells co-transfected with a plasmid expressing eGFP and either with a plasmid expressing rbFOX1, MBNL1, 960 CUG repeats or 1,000 CCUG repeats or with a siRNA directed against *rbFox1* or *Mbnl1*. **f-j** RT-PCR analysis (left panel) and quantification (right panel) of the alternative splicing of *CACNA1S* (CAV1.1), *RYR1, ATP2A1* (SERCA), *LDB3* (CYPHER/ZASP) and *MBNL1* pre-mRNAs by RT-PCR performed on total RNA extracted from adult distal skeletal muscle of controls, DM1 or DM2 individuals. Error bars indicate s.e.m. of 3 independent experiments. Student's t-test, * indicates $p<0.5$, ** indicates $p<0.01$, *** indicates $p<0.001$.

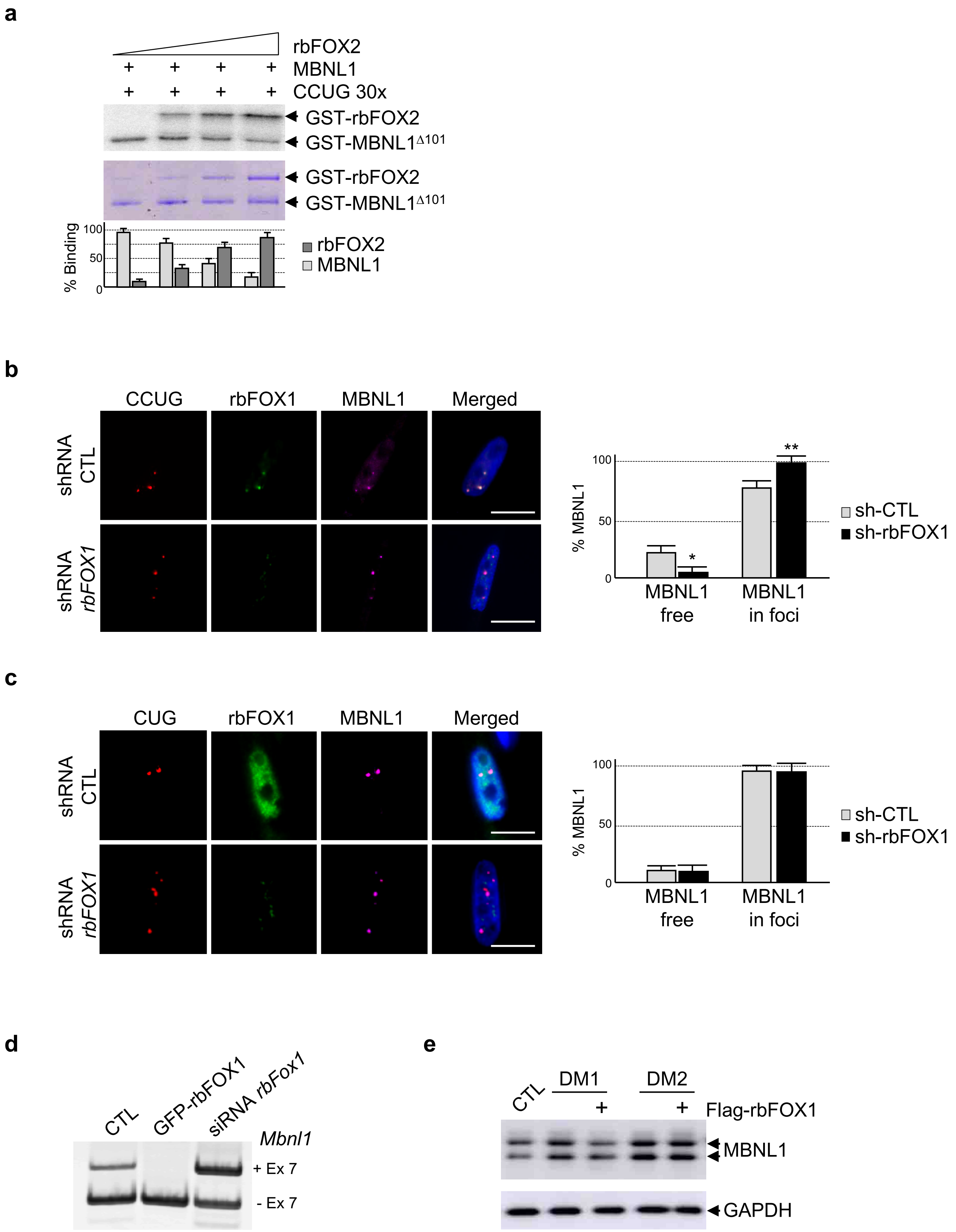

a
rbFOX2
MBNL1
CCUG 30x
GST-rbFOX2
GST-MBNL1Δ101
GST-rbFOX2
GST-MBNL1Δ101
% Binding
100
50
0
rbFOX2
MBNL1
b
CCUG
rbFOX1
MBNL1
Merged
shRNA CTL
shRNA rbFOX1
% MBNL1
100
50
0
sh-CTL
sh-rbFOX1
MBNL1 free
MBNL1 in foci
c
CUG
rbFOX1
MBNL1
Merged
shRNA CTL
shRNA rbFOX1
% MBNL1
100
50
0
sh-CTL
sh-rbFOX1
MBNL1 free
MBNL1 in foci
d
CTL
GFP-rbFOX1
siRNA rbFox1
Mbnl1
+ Ex 7
- Ex 7
e
CTL
DM1
DM2
Flag-rbFOX1
MBNL1
GAPDH

f

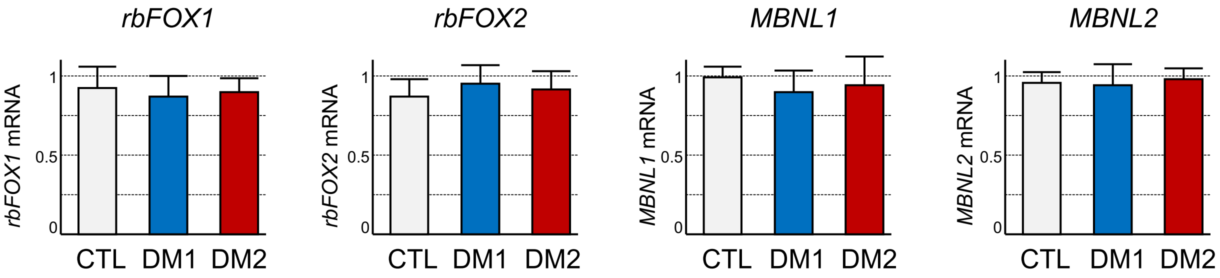
rbFOX1
rbFOX2
MBNL1
MBNL2
rbFOX1 mRNA
rbFOX2 mRNA
MBNL1 mRNA
MBNL2 mRNA
1
0.5
0
CTL DM1 DM2

g

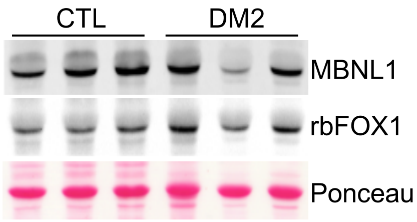
CTL
DM2
MBNL1
rbFOX1
Ponceau

h

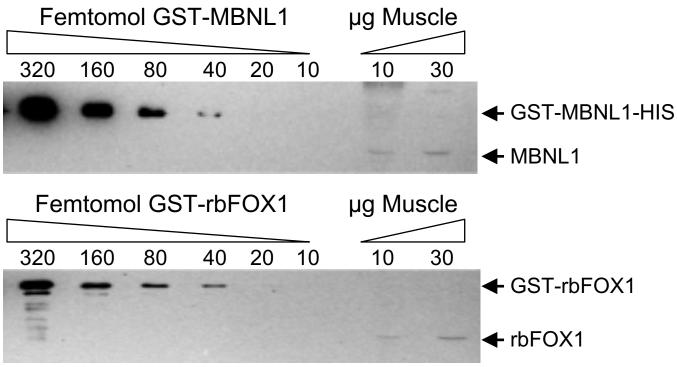
Femtomol GST-MBNL1
µg Muscle
320 160 80 40 20 10
10 30
GST-MBNL1-HIS
MBNL1
Femtomol GST-rbFOX1
µg Muscle
320 160 80 40 20 10
10 30
GST-rbFOX1
rbFOX1

Supplementary Figure 5. rbFOX1 competes with MBNL1 for binding to CCUG repeats.

a Upper panel, UV-cross-linking binding of 0.5 µg of GST-MBNL1$^{\Delta 101}$ to 10,000 CPM of RNA containing 30 CCUG repeats was competed by increasing amounts (0.25, 0.5 and 1 µg) of GST-rbFOX2. Middle panel, loading of recombinant MBNL1 and of rbFOX2 proteins was verified by coomassie staining. Lower panel, quantification of the binding of MBNL1 and rbFOX2 to expanded CCUG repeats. **b** Left panel, RNA FISH of expanded CCUG repeats coupled to the concomitant immunofluorescence of rbFOX1 and MBNL1 on primary cultures of muscle cells from individuals with DM2 transduced with an adenovirus expressing an shRNA directed against either the luciferase (CTL) vector or against *rbFOX1*. Right panel, quantification of the signal of MBNL1 either localized diffusely within the nucleoplasm (free MBNL1) or localized within the CCUG RNA aggregates (MBNL1 foci). **c** As in **b** but in primary cultures of muscle cells from individuals with DM1. **d** RT-PCR analysis of the alternative splicing of endogenous *Mbnl1* exon 7 mRNA from C2C12 cells transfected either with a plasmid expressing rbFOX1 or with a siRNA directed against *rbFox1*. **e** Immunoblot against endogenous MBNL1 expression in primary cultures of muscle cells from individuals with DM1 or DM2. **f** Quantitative RT-real time PCR analysis of the expression of *rbFOX1, rbFOX2, MBNL1* and *MBNL2* mRNAs relative to the expression to the *RPLP0* mRNA in human skeletal muscle samples of control, DM1 and DM2 individuals. **g** Immunoblotting against rbFOX1 or MBNL1 of 50 µg of control or DM2 adult muscle samples. **h** Upper panel, immunoblotting against MBNL1 of recombinant purified GST-MBNL1-HIS and of control human adult muscle samples. Lower panel, immunoblotting against rbFOX1 of recombinant purified GST-rbFOX1 and of control human adult muscle samples. Magnification, 630x. Scale bars, 10 µm. Nuclei were counterstained with DAPI. Error bars indicate s.e.m. of 3 to 5 independent experiments. Student’s t-test, * indicates $p<0.5$, ** indicates $p<0.01$.

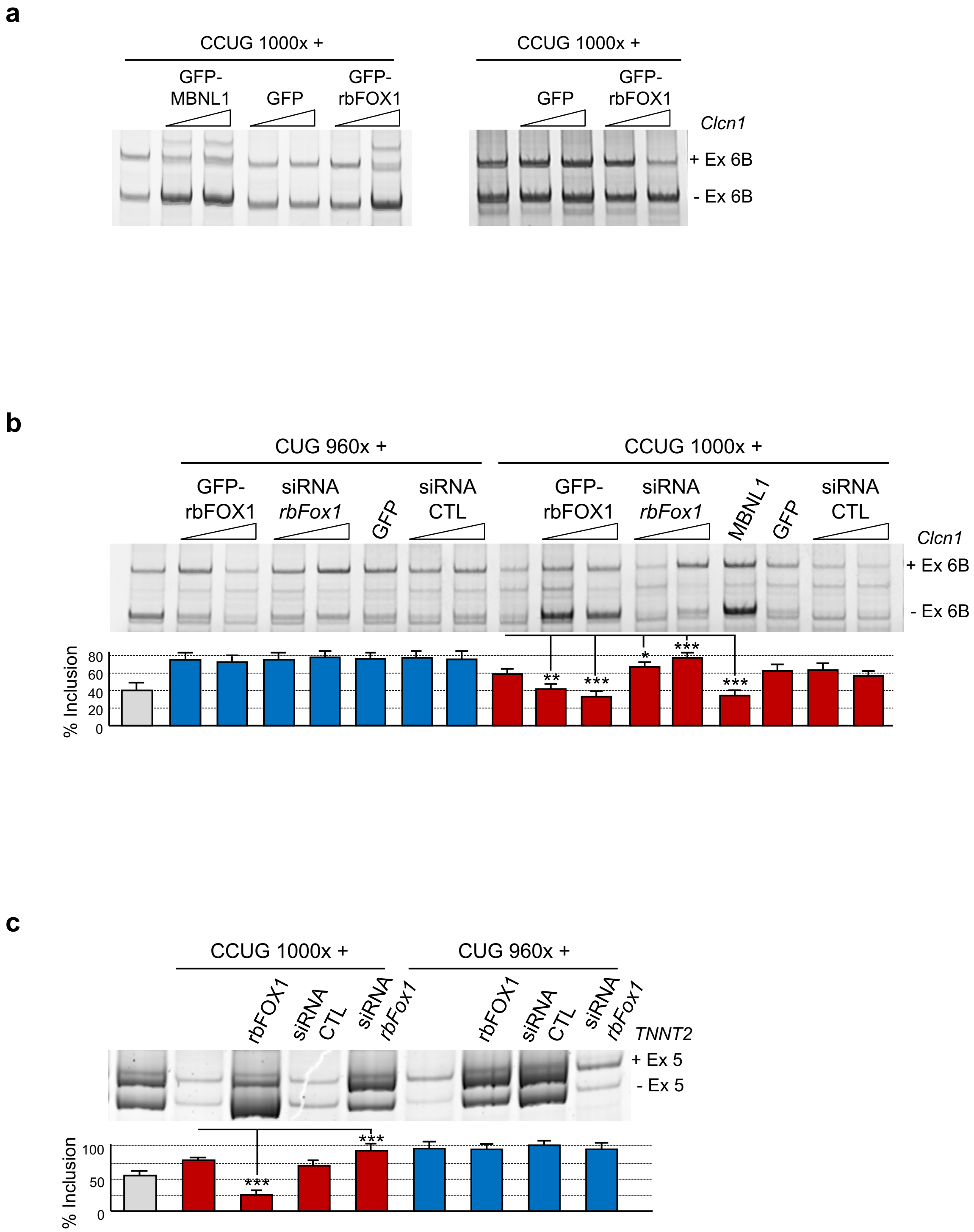
a
CCUG 1000x +
GFP-
MBNL1
GFP
GFP-
rbFOX1
CCUG 1000x +
GFP
GFP-
rbFOX1
Clcn1
+ Ex 6B
- Ex 6B
b
CUG 960x +
CCUG 1000x +
GFP-
rbFOX1
siRNA
rbFox1
GFP
siRNA
CTL
GFP-
rbFOX1
siRNA
rbFox1
MBNL1
GFP
siRNA
CTL
Clcn1
+ Ex 6B
- Ex 6B
% Inclusion
80
60
40
20
0
**
***
*
***
***
c
CCUG 1000x +
CUG 960x +
rbFOX1
siRNA
CTL
siRNA
rbFox1
rbFOX1
siRNA
CTL
siRNA
rbFox1
TNNT2
+ Ex 5
- Ex 5
% Inclusion
100
50
0
***
***

d

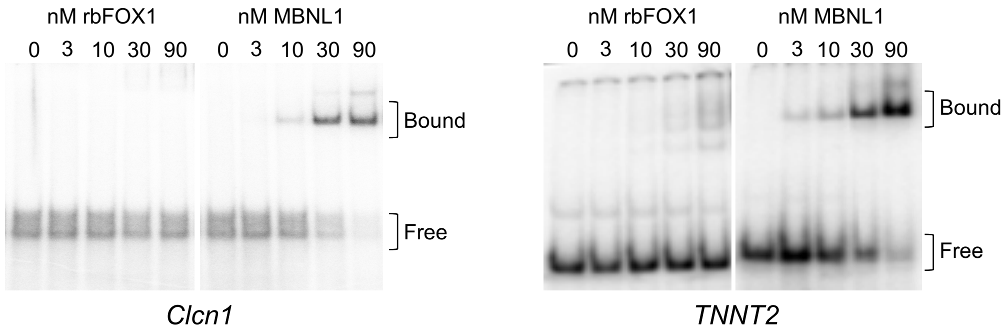

e

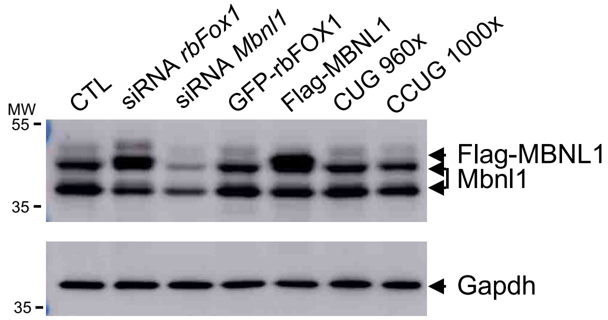

Supplementary Figure 6. rbFOX1 corrects splicing alterations caused by CCUG repeats.

a Examples of *Clcn1* exon 6B splicing analysis upon co-transfection of C2C12 muscle cells with *Clcn1* minigene, a plasmid expressing 1,000 CCUG repeats and either a vector expressing rbFOX1, MBNL1 or control GFP. **b** Upper panel, RT-PCR analysis of *Clcn1* exon 6B alternative splicing from C2C12 cells co-transfected with the *Clcn1* minigene and with a plasmid expressing either 960 CUG repeats or 1,000 CCUG repeats and either a vector expressing rbFOX1 or a siRNA targeting *rbFox1*. Lower panel, quantification of *Clcn1* exon 6B inclusion. **c** As in **a** but with a *TNNT2* exon 5 minigene. **d** Gel shift assays of 0, 3, 10, 30 and 90 nM of purified bacterial recombinant GST-rbFOX1 or GST-MBNL1$^{\Delta 101}$ with 10 pM (3,000 CPM) of RNAs corresponding to either *Clcn1* exon 6B or *TNNT2* exon 5 bordering intronic regions. **e** Immunoblot against endogenous and exogenous Mbnl1 expression from C2C12 cells co-transfected as in A. Error bars indicate s.e.m. of 3 independent experiments. Student's t-test, * indicates p<0.5, ** indicates p<0.01, *** indicates p<0.001.

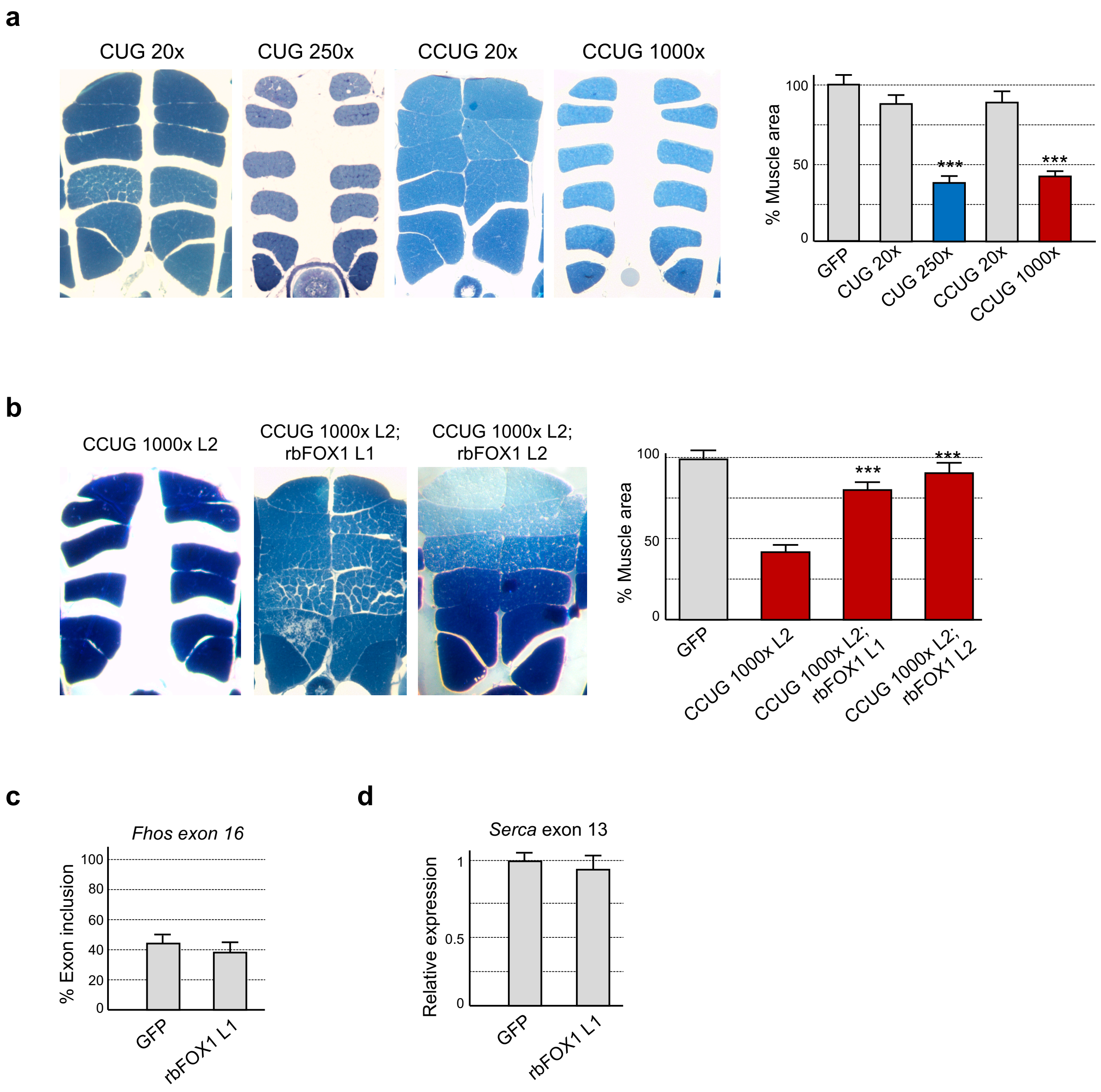
a
CUG 20x
CUG 250x
CCUG 20x
CCUG 1000x
% Muscle area
100
50
0
***
***
GFP
CUG 20x
CUG 250x
CCUG 20x
CCUG 1000x
b
CCUG 1000x L2
CCUG 1000x L2; rbFOX1 L1
CCUG 1000x L2; rbFOX1 L2
% Muscle area
100
50
0
***
***
GFP
CCUG 1000x L2
CCUG 1000x L2; rbFOX1 L1
CCUG 1000x L2; rbFOX1 L2
c
Fhos exon 16
% Exon inclusion
100
80
60
40
20
0
GFP
rbFOX1 L1
d
Serca exon 13
Relative expression
1
0.5
0
GFP
rbFOX1 L1

e

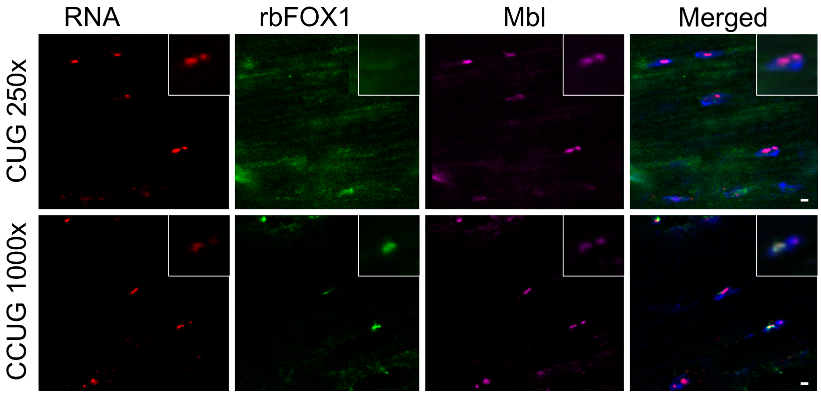

Supplementary Figure 7. rbFOX1 alleviates muscle defects in DM2 flies.

a Left panel, representative dorsoventral sections of resin-embedded adult thoraces showing indirect flight muscles (IFMs) of control non-expanded and expanded CUG or CCUG expressing flies. Right panel, IFM muscle area quantification. **b** As in **a** but with a second independent expanded CCUG expressing fly line. **c** RT-PCR quantification of endogenous *Fhos* exon 16 inclusion in flies expressing either GFP or GFP-rbFOX1. **d** Quantification of *Serca* exon 13 expression in flies expressing either GFP or GFP-rbFOX1. Detection of endogenous *Rp49* gene expression was used for normalization. **e** Representative confocal images of RNA FISH of CUG or CCUG repeats coupled to immunofluorescence of GFP-rbFOX1 using anti-GFP and anti-Mbl antibodies on rostrocaudal cryosections from adult *Drosophila* IFM of DM1 and DM2 flies. Nuclei were counterstained with DAPI (blue). Scale bars, 5µm. Error bars indicate s.e.m. of 3 independent experiments. Student's t-test, *** indicates $p<0.001$.
